# Determinants of Change in Children’s Sedentary Time

**DOI:** 10.1371/journal.pone.0067627

**Published:** 2013-06-28

**Authors:** Andrew J. Atkin, Kirsten Corder, Ulf Ekelund, Katrien Wijndaele, Simon J. Griffin, Esther M. F. van Sluijs

**Affiliations:** 1 UKCRC Centre for Diet and Activity Research (CEDAR), Institute of Public Health, Cambridge, United Kingdom; 2 Medical Research Council, Epidemiology Unit, Cambridge, United Kingdom; 3 Department of Sports Medicine, Norwegian School of Sports Sciences, Oslo, Norway; Hospital for Sick Children, Canada

## Abstract

**Background:**

Understanding the determinants of sedentary time during childhood contributes to the development of effective intervention programmes.

**Purpose:**

To examine family and home-environmental determinants of 1-year change in objectively measured sedentary time after-school and at the weekend.

**Methods:**

Participants wore accelerometers at baseline and 1 year later. Longitudinal data for after-school and weekend analyses were available for 854 (41.5%male, mean±SD age 10.2±0.3years) and 718 (41.8%male, age 10.2±0.3years) participants. Information on 26 candidate determinants, including socioeconomic status (SES), availability of electronic media and parental rules for sedentary behaviours was self-reported by children or their parents at baseline. Change in the proportion of registered time spent sedentary was used as the outcome variable in multi-level linear regression models, adjusted for age, sex, body mass index and baseline sedentary time. Simple and multiple models were run and interactions with sex explored.

**Results:**

Children from higher socioeconomic status families exhibited greater increases in after-school (beta; 95% CI for change in % time spent sedentary 1.02; 0.37, 1.66) and weekend (1.42; 0.65, 2.18) sedentary time. Smaller increases in after-school sedentary time were observed in children with more siblings (−1.00; −1.69, −0.30), greater availability of electronic media (−0.81; −1.29, −0.33) and, for boys, more frequent family visits to the park (−1.89; −3.28, −0.51) and family participation in sport (−1.28; −2.54, −0.02). Greater maternal weekend screen-time (0.45; 0.08, 0.83) and, in girls, greater parental restriction on playing outside (0.91; 0.08, 1.74) were associated with larger increases in weekend sedentary time. The analytical sample was younger, more likely to be female, had lower BMI and was of higher SES than the original baseline sample.

**Conclusions:**

Intervention strategies aimed at reducing parents’ weekend screen-time, increasing family participation in sports or recreation (boys) and promoting freedom to play outside (girls) may contribute towards preventing the age-related increase in sedentary time.

## Introduction

In the contemporary epidemiological literature, sedentary behaviours are conceptualised as being behaviourally distinct from the absence of moderate-to-vigorous physical activity (MVPA).[1]–[4] As such, this group of behaviours, which includes television (TV) viewing and travelling by motorised transport, may present a health risk that is independent of MVPA. During childhood, these highly prevalent behaviours, which appear to increase with age [5]–[8], may be associated with adiposity, low fitness, some cardiovascular disease risk factors and poorer mental health, [9]–[12] though the evidence is not wholly consistent. [13] Whilst further longitudinal and experimental research is required to clarify the role of sedentary behaviours as an independent health risk factor, there remain strong grounds for examining sedentary behaviour in a public health context. The time available each day for children to engage in sedentary and physically active behaviours is fixed and finite. Previous research has demonstrated that changes in children’s screen or social sedentary behaviour may impact upon time allocated to sleep and physical activity. [14], [15] Therefore, knowledge of the determinants of sedentary behaviour may contribute towards the promotion of physical activity, by enabling the development of intervention strategies to shift children’s behaviour from sedentary to more active pursuits. Public health guidelines in the UK recommend that young people should minimise the amount of time spent being sedentary for prolonged periods. [16].

To inform the development of intervention programmes, it is necessary to identify population groups at risk of high levels of sedentary behaviour and modifiable factors that can be targeted to reduce participation. [17] Informed by an ecological model of health behaviour, it may be hypothesised that factors operating at individual, social, and environmental levels may influence children’s sedentary behaviour patterns, though few studies attempt to examine the relative influence of factors from multiple levels simultaneously. [18] To date, family and home-environmental characteristics, such as parental sedentary behaviours, availability of electronic media and parental rules, consistently have been associated with children’s sedentary behaviour patterns.[19]–[22] However, much of this evidence is drawn from cross-sectional studies using self-report measures of TV viewing or other screen-based behaviours [23]–[25]; such measures fail to capture the diversity and entirety of children’s sedentary behaviour.[23]–[28] In order to limit or reduce children’s overall sedentary time, as recommended in public health guidelines, [16] studies examining the determinants of total sedentary time are required. Prospective studies examining the determinants of children’s objectively measured sedentary time are lacking. [7], [29], [30] Therefore, the aim of the current study was to examine the association of social, behavioural and environmental characteristics of the home and family with changes in children’s objectively-measured non-school sedentary time over 1 year. To ascertain whether tailored intervention approaches may be necessary for boys and girls, we tested for effect modification by sex in our statistical models. We acknowledge that numerous moderators may exist (e.g. weight status, ethnicity) but felt it was not feasible to examine multiple moderators within the scope of the current analysis. We focus upon sex because previous research indicates that the correlates of sedentary and physically active behaviours may differ for boys and girls [19]–[23], [30] but few studies test for such interactions statistically.

## Methods

### Design and Ethics Statement

The Sport, Physical Activity, and Eating Behaviour: Environmental Determinants in Young People (SPEEDY) study is a population based cohort study investigating factors associated with physical activity, sedentary behaviour and diet in children from the county of Norfolk, UK. [31] Ethical approval was obtained from the University of East Anglia research ethics committee.

### Data Collection Procedures

Full details of participant recruitment and procedures for baseline data collection have been reported previously. [31] Of the 157 schools approached to participate in SPEEDY, 92 (response rate 58.6%) were visited for measurement. At participating schools, all children in school year 5 (N = 3619) and their parents were sent an invitation pack. In total, 2064 children provided parental consent and were measured at baseline (57.0% response rate). Baseline data collection took place during the school term, between April-July 2007. Trained research assistants visited schools to take physical measurements, administer child questionnaires, fit accelerometers, and distribute a home pack (containing an accelerometer diary, instruction sheet, questionnaire, and food diary). Participants were requested to return the home pack one week later.

Follow-up data collection took place 1 year later (April–July 2008). Study information sheets and consent forms were mailed to all 2064 initial participants. Those who consented were mailed an accelerometer and a detailed instruction sheet. Participants were asked to wear the accelerometer for one week and to return it by mail, using an addressed, pre-paid envelope. Individual participants were measured at approximately the same time of year as at baseline.

### Sedentary Behaviour Measurement

Sedentary time was measured objectively using an Actigraph (GT1M; Pensacola, FL) accelerometer [32], [33], set to record at 5-second epochs. Children were instructed to wear the monitor during waking hours for 7 days and to remove it while bathing, showering and swimming. Accelerometer data were analysed using a batch processing program (MAHUffe; http://legacy.mrc-epid.cam.ac.uk/Research/Programmes/Programme_5/InDepth/Programme%205_Disclaimer.html). A count threshold of <100 counts per minute (cpm) was used to define sedentary time. [34], [35] Periods of ≥10 minutes of consecutive zero counts [36], [37] and days with <500 minutes of recording between 6 am–11 pm were excluded. [29], [36] Two sedentary time outcome variables were derived and analysed separately; (1) after-school (3–11 pm, Monday-Friday) and (2) at the weekend (6 am–11 pm, Saturday/Sunday). To account for differences in accelerometer wear time between baseline and follow-up, outcome variables were constructed as change in the proportion of time spent sedentary, calculated as follows: [(follow-up sedentary time/follow-up wear time)×100]–[(baseline sedentary time/baseline wear time)×100]. A minimum of 2 days of weekday data and 1 day of weekend data was required for inclusion in the after-school and weekend analyses respectively. Change in sedentary time (min/day) between baseline and follow-up was estimated by multiplying the proportion of time spent sedentary by the mean wear time for the appropriate time period.

### Family and Home-environmental Factors

Twenty six determinants were included in the analysis, grouped under the following headings: socio-demographic, parent behaviours, family rules and activities, home environment (Table 1). Data on putative determinants were self-reported by children or their parents at baseline using previously tested items where possible. All determinants were assessed using a single item, except for sedentary behaviour restriction (3 items; Cronbach’s α 0.8) and indoor play rules (2 items; Cronbach’s α 0.6). Due to lack of heterogeniety (>90% of responses in one category or direction), ethnicity (96.8% white), whether or not there was a garden at home (98.9% yes), and whether or not there was a TV (99.8% yes) or computer (95.7% yes) at home were not included in the analyses.

**Table 1:** Description of family and home environment determinants.

Variable name^a^	Description and/or coding
Socio-demographics (Parent-reported)	
Socioeconomic status	Composite score (range 0–3) calculated as the sum of 3 items: Age main caregiver left full-time education (≤16 years coded as 0, >16 years coded as 1); car ownership (‘no’ coded as 0, ‘yes’ coded as 1); home ownership (renting coded as 0, own/buying coded as 1).
Index of Multiple Deprivation (IMD)	Derived from caregiver reported home postcode at baseline. The IMD is a tool used for ranking area-level deprivation in England. [53] Analysed in quartiles (quartile 1 = least deprived).
Parents at home	Number of parents living at home. Coded as 1 or 2.
Siblings	Number of siblings living at home. Coded as 0, 1, 2 or more.
Urban/rural	Home located in rural/urban location (rural coded as 0, urban coded as 1). Derived from home postcode using methods described by Bibby and Shephard. [54] Four density profiles were collapsed into a dichotomous variable; ‘city’/‘town and fringe’ classified as urban, ‘hamlets and isolated dwellings’/‘villages’ classified as rural.
Lives in a cul-de-sac	Family home is located in a cul-de-sac (non-through road) (‘no’ coded as 0, ‘yes’ coded as 1), derived using Geographical Information Systems data.
**Parent behaviours (Parent-reported)**	
Mother/Father weekday TV viewing and computer use [55]	Composite score (range 2–12) calculated as sum of responses to 2 items on time spent TV viewing or using a computer outside of work on weekdays. Individual items had 6 response options (none, <1 hr/day, 1–2 hr/day, 2–3 hr/day, 3–4 hr/day 4+ hr/day).
Mother/Father weekend TV viewing and computer use [55]	Composite score (range 2–12) calculated as sum of responses to 2 items on time spent TV viewing or using a computer outside of work on weekend days. Individual items had 6 response options (none, <1 hr/day, 1–2 hr/day, 2–3 hr/day, 3–4 hr/day 4+ hr/day).
Mother/Father physical activity [56]	Previously validated index based on occupational and leisure-time physical activity. Coded as inactive, moderately inactive, moderately active, active.
**Family rules and activities (Parent-reported)**	
Sedentary behaviour restriction [57], [58]	Composite score (range 3–15) calculated as the sum of 3 items: frequency that caregivers restrict TV viewing, playing computer games, using a computer. Individual items had 5 response options (never, rarely, sometimes, often, very often) (Cronbach’s α 0.8)
Playing outside restriction [57]	Frequency that caregivers restrict the child from playing outside. Five response options (never, rarely, sometimes, often, very often)
Indoor play rules [57], [58]	Composite score (range 2–8) calculated as sum of 2 items: frequency that caregivers allow child to run around the house/play ball games in the house. Individual items had 4 response options (never, rarely, sometimes, often/very often) (Cronbach’s α 0.6)
Neighbourhood play rules [57]	Frequency that caregivers allow child to play outside in the neighbourhood. Four response options (never, rarely, sometimes, often/very often).
	
Playing after dark rules [57]	Frequency that caregivers allow child to play outside after dark. Four response options (never, rarely, sometimes, often/very often).
Bedtime rules [57]	Frequency that caregivers allow child to go to bed when they want to. Four response options (never, rarely, sometimes, often/very often).
Reading as a family [57]	Weekly frequency of reading together as a family (never, 1–4 times/week, >4 times/week)
Watching TV as a family [57]	Weekly frequency of watching television together as a family (never, 1–4 times/week, >4 times/week)
Playing sport as a family [57]	Weekly frequency of playing sport together as a family (never, 1–4 times/week, >4 times/week)
Visiting relatives as a family [57]	Weekly frequency of visiting friends/relatives as a family (never, 1–4 times/week, >4 times/week)
Going to the park as a family [57]	Weekly frequency of going to the park as a family (never, 1–4 times/week, >4 times/week)
**Home environment (Child-reported)**	
Shared bedroom	Participant shares a bedroom with a sibling. (‘no’ coded as 0, ‘yes’ coded as 1)
Games console [59]	Games console at home. (‘no’ coded as 0, ‘yes’ coded as 1)
Electronic equipment in bedroom [59]	Composite score (range 0–3) calculated as the sum of 3 items: presence of TV, games console, or desktop computer in bedroom (‘no’ coded as 0, ‘yes’ coded as 1).

a References are provided to the source of questions where applicable.

Where appropriate, there lowest coded group was used as the reference group.

### Statistical Analysis

Analyses were conducted using Stata (version 11.0) in 2012. We compared baseline characteristics among those included and lost to follow-up using Student’s *t* tests and *Χ* ^2^ tests. Accounting for school-level clustering, multi-level (random intercept) linear regression was used to test for differences in sedentary time between baseline and follow-up. Separately for after-school and weekend outcome variables, multi-level (random intercept) linear regression was used to examine associations of putative determinants assessed at baseline with change in the proportion of time spent sedentary from baseline to follow-up. The intra-class correlations (ICC) for change in after-school and weekend sedentary time were 0.01 and 0.07 respectively. Determinants were coded as binary or ordered categorical variables. Ordered categorical variables were linearly associated with outcome measures and therefore treated as continuous in statistical models. Initially, simple associations between determinants and sedentary time outcomes were examined, with adjustment for baseline level of the outcome variable only. [38] Subsequently, interaction terms were added to regression models to explore effect modification by sex. Determinants, and associated interaction terms, with *P*<0.1 in simple models were entered into a single multivariable model. Multivariable models were adjusted for age, sex, BMI, and baseline level of the outcome variable. In the multivariable model, variables with *P*>0.05 were sequentially removed, one at a time starting with the highest *P*-value, to derive the ‘final model’.

Information on father characteristics was not available for those households where a (step-) father was not present in the home (after-school analysis *N* = 127, weekend analysis *N* = 104). To avoid potential selection bias, father characteristics that were significantly associated in simple models were only entered into ‘final models’ to determine whether they remained significant after adjustment for potential confounders and other significant determinants.

## Results

Of the 2064 participants from baseline measurement that were invited to take part in follow-up, 1019 (49.4% of the baseline sample) provided parental consent. Of those participants, 954 (46.2% of baseline sample; 93.6% of follow-up sample) returned an activity monitor containing data. Valid accelerometer data on changes in after-school and weekend sedentary time was obtained for 854 (41.4% of baseline sample; 83.8% of follow-up sample) and 718 (34.8% of baseline sample; 70.5% of follow-up sample) participants, respectively. Participants included in the analyses were younger (after-school *P* = 0.02; weekend *P* = 0.04), more likely to be female (after-school *P* = 0.009; weekend *P* = 0.04), had lower BMI (after-school *P* = 0.003; weekend *P* = 0.01), and were more likely to be of higher SES (after-school and weekend *P*<0.001) than those from the baseline sample (n = 2064) who did not provide outcome data at follow-up. Table 2 presents baseline characteristics for 854 participants providing valid data on after-school sedentary time at baseline and follow-up. Over 1 year, sedentary time increased both after-school and at the weekend (Table 3).

**Table 2:** Baseline characteristics of participants with valid accelerometer data at baseline and follow-up (N = 854).

	All	Girls	Boys
**Gender, n (%)**	854 (100)	500 (58.5)	354 (41.5)
**Age, mean ± SD, y**	10.2±0.3	10.2±0.3	10.2±0.3
**Proportion owning/buying home, %**	79.4	77.1	82.6
**Proportion owning car, %**	96.8	97.0	96.5
**Proportion of mothers who left full time education >16y, %**	56.1	55.3	57.4
**Composite SES, %**			
** Lowest (score: 0 or 1)**	14.1	15.3	12.4
** Middle (score: 2)**	37.5	38.2	36.6
** Highest (score: 3)**	48.4	46.5	51.0
**After-school** a **sedentary time, mean ± SD, min/d**	198.2±38.6	199.9±38.3	195.8±38.9
**Weekend sedentary time mean ± SD, min/d**	430.8±72.2	433.9±71.0	426.5±73.7
**After-school** a **wear time, mean ± SD, min/d**	319.3±51.5	318.2±52.6	320.9±49.8
**Weekend wear time, mean ± SD, min/d**	691.9±82.4	685.3±82.0	701.2±82.2

SD, standard deviation; SES, socioeconomic status.

Wear time at follow-up (mean ± SD) min/d: After-school All = 320.6±57.0, Girls = 321.4±56.4, Boys = 319.4±57.8; Weekend All = 683.7±82.1, Girls = 679.9±77.7, Boys = 688.9±87.6.

a After-school defined as 3–11pm Monday to Friday.

**Table 3:** One-year change in accelerometer-assessed sedentary time after school and at the weekend.

	After school		Weekend	
	(N = 854;500 girls, 354 boys)		(N = 718; 418 girls, 300 boys)	
	Change	*P*	Change	*P*
**Sedentary time, mean ± SD, % of day**				
**All**	1.3±7.8	0.001	2.1±8.9	<0.001
**Girls**	1.2±7.6	<0.001	1.8±8.9	<0.001
**Boys**	1.5±8.1	<0.001	2.5±8.9	<0.001
**Sedentary time, mean, min/d** a				
**All**	4.2	0.001	14.4	<0.001
**Girls**	3.8	<0.001	12.5	<0.001
**Boys**	4.7	<0.001	17.1	<0.001

a Estimates of change in sedentary time calculated as proportion of period spent sedentary multiplied by outcome-specific mean wear time (After school mean wear time = 319.9 min; Weekend mean wear time = 689.9 min).

### Determinants of Change in After-school Sedentary Time

Simple associations between determinants and change in the proportion of time spent sedentary after-school and at the weekend are presented in Table 4. Eleven variables were significantly associated with change in after-school sedentary time and were carried forward to the multivariable model. Six determinants were independently associated with change in after-school sedentary time in the final model (Table 5). Children from higher SES families recorded greater increases in sedentary time compared to those of low SES. Children with a greater number of siblings and those with more electronic media in the bedroom exhibited smaller increases in time spent sedentary. For boys only, more frequent episodes of playing sport or visiting the park as a family were associated with smaller increases in sedentary time. A significant interaction with sex was observed for frequency of watching television as a family; in stratified analysis, however, associations in both boys and girls were non-significant.

**Table 4:** Associations for change in the proportion of time spent sedentary with family and home-environmental determinants.

Variable	After-school	Weekend
	β Coefficient (95% CI)	β Coefficient (95% CI)
**Socio-demographics**		
Socioeconomic status	**1.20 (0.57, 1.82)** ***	**1.16 (0.42, 1.90)** **
Index of Multiple Deprivation	−0.23 (−0.66, 0.20)	−0.31 (−0.84, 0.21)
Parents at home	0.55 (−0.73, 1.83)	1.25 (−0.28, 2.77)
Siblings	−**0.87 (**−**1.56,** −**0.19)** *	0.37 (−0.44, 1.18)
Urban/rural	−0.39 (−1.38, 0.61)	0.09 (−1.12, 1.30)
Lives in a cul-de-sac	0.02 (−0.97, 1.00)	0.12 (−1.04, 1.29)
**Parent behaviours**		
Mother weekday TV and computer use	0.18 (−0.17, 0.52)	0.18 (−0.22, 0.58)
Mother weekend TV and computer use	0.14 (−0.17, 0.46)	**0.48 (0.1, 0.85)** *
Mother physical activity	−0.17 (−0.62, 0.29)	−0.31 (−0.83, 0.22)
**Family rules and activities**		
Sedentary behaviour restriction	0.02 (−0.14, 0.19)	0.03 (−0.17, 0.22)
Playing outside restriction	**0.51 (**−**0.06, 1.09)** †	**0.32 (**−**0.35, 0.98)^‡^**
Indoor play rules	0.13 (−0.16, 0.42)	0.16 (−0.18, 0.50)
Neighbourhood play rules	0.07 (−0.32, 0.46)	0.10 (−0.36, 0.55)
Playing after dark rules	0.41 (−0.23, 1.04)	−0.07 (−0.82, 0.68)
Bedtime rules	**0.54 (**−**0.06, 1.14)** †	**0.49 (**−**0.22, 1.21)^‡^**
Reading as a family	−**0.39 (**−**1.09, 0.31)^‡^**	−0.42 (−1.24, 0.41)
Watching TV as a family	−**0.01 (**−**0.89, 0.86)^‡^**	0.16 (−0.87, 1.18)
Playing sport as a family	−**0.41 (**−**1.22, 0.40)^‡^**	−0.65 (−1.63, 0.32)
Visiting relatives as a family	0.33 (−0.78, 1.44)	−0.60 (−1.90, 0.70)
Going to the park as a family	−**0.85 (**−**1.71, 0.02)** † **^‡^**	−**0.79 (**−**1.79, 0.21)^‡^**
**Home environment**		
Shared bedroom	−**1.26 (**−**2.39,** −**0.14)** *	0.05 (−1.28, 1.39)
Games console	−**1.79 (**−**3.19,** −**0.39)** *	−0.40 (−2.04, 1.24)
Electronic equipment in bedroom	−**0.86 (**−**1.32,** −**0.41)** ***	−**0.67 (**−**1.21,** −**0.13)** *

Variables in **bold** carried forward to the multi-variable model.

95% CI, 95% confidence interval.

† *P*<0.1;

* *P*<0.05;

** *P*<0.01;

*** *P*<0.001; ‡interaction with sex (*P*<0.1).

Numbers for the after-school (*N* = 806–854) and weekend (*N* = 678–718) analyses varied due to missing data for individual determinants.

**Table 5:** Final multivariable models for association of family and home-environmental determinants with change in sedentary time.

	β Coefficient (95% CI)
**After-school**	
Socioeconomic status	1.02 (0.37, 1.66)**
Siblings	−1.00 (−1.69, −0.30)**
Watching TV as a family (boys)	1.29 (−0.06, 2.64)
* Sex* * *Watching TV as a family* a	−*1.94 (*−*3.71,* −*0.18)* *
Playing sport as a family (boys)	−1.28 (−2.54, −0.02)*
* Sex* * *Playing sport as a family* b	*1.77 (0.08, 3.47)* *
Going to the park as a family (boys)	−1.90 (−3.28, −0.51)**
* Sex* * *Going to the park as a family* c	*2.41 (0.62, 4.19)* **
Electronic equipment in bedroom	−0.81 (−1.29, −0.33)*
**Weekend**	
Socioeconomic status	1.42 (0.65, 2.18)***
Mother weekend TV and computer use	0.45 (0.08, 0.83)*
Playing outside restriction (boys)	−1.05 (−2.18, 0.08)
* Sex* * *Playing outside restriction* d	*1.96 (0.57, 3.36)* **

Sex-specific associations obtained from full model.

a β Coefficient (95% CI) for association in girls −0.66 (−1.80, 0.49).

b β Coefficient (95% CI) for association in girls 0.49 (−0.64, 1.62).

c β Coefficient (95% CI) for association in girls 0.51 (−0.64, 1.66).

d β Coefficient (95% CI) for association in girls 0.91 (0.08, 1.74)*.

95% CI, 95% confidence interval.

* *P*<0.05;

** *P*<0.01;

*** *P*<0.001.

### Determinants of Change in Weekend Sedentary Time

In simple models, 6 variables were associated with change in weekend sedentary time and were included in the multivariable model. Three variables were retained in the final model. Children from higher SES families recorded greater increases in sedentary time compared to those of low SES. Children whose mothers spent more time TV viewing/using a computer at the weekend showed greater increases in sedentary time. More frequent restriction on playing outside was associated with greater increases in girls’ sedentary time.

### Father-level Determinants of Change in Sedentary Time

Among participants with a (step-) father living at home, father’s TV viewing and computer use on weekdays (beta; 95%CI; *P*: 0.34; 0.02, 0.66; *P* = 0.04) and at the weekend (0.26; −0.03, 0.56; *P* = 0.08) were positively associated with change in sedentary time after-school. Interactions with sex were observed for father’s physical activity (Boys; 0.69; −0.08, 1.46; Girls −0.47; −1.10, 0.16; *P for interaction = *0.02) and weekend TV viewing and computer use (Boys; −0.07; −0.52, 0.38; Girls 0.50; 0.12, 0.89; *P for interaction = *0.06). When these variables were added to the final model derived in the full sample, one significant association remained; father’s physical activity was positively associated with change in boys after-school sedentary time (1.34; 0.53, 2.15; *P* = 0.001).

In simple models, father’s weekend TV viewing and computer use (beta; 95%CI; *P*: 0.52; 0.19, 0.86; *P* = 0.002) was positively associated and father’s physical activity (−0.55; 0. −1.12, 0.02; *P* = 0.06) negatively associated with change in sedentary time at the weekend. No significant interactions with sex were observed. When added to the final model derived in the full sample, the positive association between father’s TV viewing and computer use at the weekend remained significant (0.39; 0.01, 0.78; *P* = 0.04).

## Discussion

Over 1 year, small increases in children’s sedentary time after-school and at the weekend were observed (Table 3). Children from higher SES families exhibited greater increases in sedentary time compared to those of lower SES (Table 5). Familial-level factors, such as parent’s weekend TV viewing and computer use, whole-family participation in sport or recreation, and rules regarding playing outside, might be targeted to prevent or reduce the age-related increase in sedentary time. Identified determinants were often sex- or time-specific, suggesting that tailored intervention strategies, focusing upon particular periods of the week or gender groups, may be necessary (Table 5).

### Comparison with Other Evidence

Existing studies of the determinants of change in children’s objectively measured sedentary time have focussed predominantly upon anthropometric or socio-demographic factors. [7], [29], [39] Familial-level characteristics, including playing sport as a family and having restrictions on outside play, have been linked to screen-based sedentary behaviours and physical activity in children [22], [40] but, to our knowledge, this is the first evidence that such characteristics may be associated with changes in children’s overall (non-school) sedentary time. Findings support the application of family-based strategies to modify sedentary behaviours in children, though verification in future studies is required.

In this study, children from higher SES families exhibited greater increases in sedentary time than those of lower SES. This represents a broadening of socio-economic differences, as SES was positively associated with sedentary time at baseline (data not shown). Mitchell et al [7] also reported that higher maternal education was associated with greater increases in British children’s sedentary time from 12–16 years of age. However, no association between maternal education and 3-year change in sedentary time was observed in Australian children (age 10–12 years at baseline) [39] and change in children’s screen-based sedentary behaviour may be inversely associated with parental education. [41], [42] Currently, the socioeconomic patterning of change in sedentary behaviour during childhood remains unclear; however, it appears that the association may vary dependent upon how these constructs are defined and measured. That said, in sensitivity analyses, the direction of the association between SES and change in sedentary time remained unchanged in models using parents educational attainment and each of our component markers of SES individually (data not shown). Future work exploring how socioeconomic factors influence engagement in specific activities may help to identify behavioural patterns that underlie the associations observed in this study.

Mother’s and (where applicable) father’s weekend TV viewing and computer use was positively associated with change in children’s weekend sedentary time. In cross-sectional studies, children’s screen-based sedentary behaviour is positively associated with that of their parents,[20]–[22] but evidence from prospective studies is inconclusive. Davison et al [43] found that mother’s TV viewing at baseline was positively associated with their daughter’s viewing habits 2 years later, but there was no association between changes in mothers and daughters viewing habits over the same period. In the same study, father and daughter viewing patterns were positively associated in cross-sectional but not longitudinal analyses. In this study, and others on this subject, parental sedentary behaviours were assessed using self-report methods. Error in the measurement of this exposure may have contributed towards the null findings reported in previous research and led to underestimation of the association in the current analysis. Clarification of the role that caregiver modelling plays in shaping the sedentary behaviour of children requires that consideration is given to all caregivers in the household and acknowledgement that influences may vary between different family structures. It is necessary to investigate how parental behaviour influences overall sedentary time and whether this association is different to that seen for specific sedentary behaviours. Studies that jointly assess specific sedentary behaviours and total sedentary time are required.

Unexpectedly, we found that children with more electronic media in their bedroom at baseline exhibited smaller increases in sedentary time. The proportion of participants with 0, 1, 2, or 3 items of electronic media in their bedroom was 25.0, 27.0, 35.1 and 12.9% respectively. Cross-sectional research typically has shown that having electronic media in the bedroom is associated with greater screen-based sedentary behaviour. [44], [45] However, previous research has failed to consistently identify a positive association between bedroom media and overall sedentary time. [46], [47]; this was also the case in cross-sectional analysis of data conducted as part of the current study (data not shown). A possible explanation for this counter-intuitive finding is that our analysis was based upon baseline exposures only, but the availability of electronic devices in the bedroom may have changed between baseline and follow-up. Thus, relative to children with more electronic media in their bedroom at baseline, children who acquired new electronic devices between baseline and follow-up may have experienced greater increases in sedentary time. As an intervention strategy, the impact of removing electronic media from children’s bedrooms has been little studied. [48] However, this strategy may create conflict between parents and children [49]; alternative approaches, perhaps focusing on parenting rules or limit setting, may be preferable. Research exploring how the availability of electronic media in the bedroom changes during childhood and how this impacts upon sedentary habits will provide valuable information for intervention design.

### Implications

There is a need to better understand the determinants of sedentary time and to develop and evaluate interventions to reduce or minimise the observed increase with age. Modifiable determinants identified in this study were often sex- or time-specific. For example, boys from families that more frequently visited the park exhibited smaller increases in sedentary time after-school; girls with more restrictions on playing outside showed greater increases in weekend sedentary time. These context- and sex-specific associations should be acknowledged in future research. The influence of family- and home-environmental factors on children’s sedentary behaviour is a consistent feature of the correlates literature and strongly supports the application of family-level strategies within intervention programmes.

To date, interventions aimed at reducing sedentary behaviour in children have produced small, but significant, changes; [48], [50], [51] however, these studies typically have focussed on a limited range of behaviours and it is generally not clear how time was reallocated if a reduction in the targeted behaviour was achieved. Our findings highlight a number of modifiable determinants that may be targeted to bring about changes in children’s overall (non-school) sedentary time, potentially providing greater net benefit than those focussed upon selected behaviours. The associations observed in this study were typically small in magnitude. For example, increases in after-school sedentary time were approximately 5–10 minutes greater in children from higher SES families compared to those from lower SES groups. However, when accumulated across the entire week and considered alongside other determinants identified in this study, the impact upon children’s sedentary time may be substantial.

### Strengths and Limitations

A key strength of this study is the use of accelerometry to assess sedentary time in a large population based cohort of children. To ensure specificity between exposure and outcome measures, analyses were restricted to periods of the day and week when family and home-environmental factors most plausibly will influence sedentary behaviour patterns. Reflective of the exploratory nature of the study, a wide variety of exposures were examined, increasing the likelihood of identifying potential determinants and enabling better control for confounding. However, a large number of statistical tests were conducted; the possibility that some of the associations are chance findings cannot be ruled out. Associations highlighted in this study require further investigation and confirmation. Total (non-school) sedentary time is comprised of numerous sedentary behaviours, which may themselves have different determinants; this may have resulted in masking of some associations. Data were collected in 2007–2008 and it is possible that behaviour patterns may have changed since this time. However, it is unlikely that the familial associations underpinning behaviour have changed substantively during this period. There was evidence of selective drop-out, by age, sex, BMI and SES between baseline and follow-up, potentially limiting the generalisability of findings. Reduced heterogeneity in our SES exposure variable, as a result of differential drop-out, may have resulted in an underestimation of the association with change in sedentary time. In addition, the demographic and socioeconomic make-up of the SPEEDY sample may not be representative of the broader UK population. Lastly, no consensus exists for the processing of accelerometer data, for example in the choice of count threshold applied. The correlation between sedentary time estimated using 100 versus 200 cpm cut-points was 0.99, suggesting that the results of the associations examined are unlikely to be affected by the threshold applied. We adopted a conservative non-wear criterion of 10 minutes of consecutive zero counts, in order to minimise potential misclassification of non-wear time as sedentary time. We acknowledge the potential for underestimation of sedentary time as a result of this approach, though this effect is likely to be relatively minor. [52].

### Conclusion

In children aged 10 years, significant increases in objectively measured sedentary time after-school and at the weekend were observed over 1 year; greater increases were noted amongst children from higher SES families. A number of potentially modifiable determinants of change in sedentary time were identified, highlighting features of the family and home-environment that could be targeted within intervention programmes. Further studies that examine the association between the presence of electronic media in the bedroom and change in children’s sedentary time are required. Reducing parents’ weekend screen-time, increasing family participation in sports or recreation (for boys) and promoting freedom to play outside (for girls) may be beneficial in efforts to prevent or reduce the age-related increase in children’s sedentary time.
